# Human acellular amniotic membrane incorporating exosomes from adipose-derived mesenchymal stem cells promotes diabetic wound healing

**DOI:** 10.1186/s13287-021-02333-6

**Published:** 2021-04-29

**Authors:** Shune Xiao, Chunfang Xiao, Yong Miao, Jin Wang, Ruosi Chen, Zhexiang Fan, Zhiqi Hu

**Affiliations:** 1 Department of Plastic Surgery, Nan Fang Hospital, Southern Medical University, Guangzhou Da Dao Bei 1838, Guangzhou, 510515 China; 2 Department of Plastic Surgery, Affiliated Hospital of Zunyi Medical University, Zunyi, Guizhou China; 3 Department of Obstetrics and Gynecology, Nan Fang Hospital, Southern Medical University, Guangzhou, Guangdong China

**Keywords:** Adipose-derived mesenchymal stem cells, Exosomes, Diabetic wound healing, Acellular amniotic membrane

## Abstract

**Background:**

Diabetic wounds threaten the health and quality of life of patients and their treatment remains challenging. ADSC-derived exosomes have shown encouraging results in enhancing diabetic wound healing. However, how to use exosomes in wound treatment effectively is a problem that needs to be addressed at present.

**Methods:**

A diabetic mouse skin wound model was established. ADSC-derived exosomes (ADSC-Exos) were isolated, and in vitro application of exosomes was evaluated using human umbilical vein endothelial cells (HUVECs) and human dermal fibroblasts (HDFs). After preparation and characterization of a scaffold of human acellular amniotic membrane (hAAM) loaded with ADSC-Exos in vitro, they were transplanted into wounds in vivo and wound healing phenomena were observed by histological and immunohistochemical analyses to identify the wound healing mechanism of the exosome-hAAM composites.

**Results:**

The hAAM scaffold dressing was very suitable for the delivery of exosomes. ADSC-Exos enhanced the proliferation and migration of HDFs and promoted proliferation and tube formation of HUVECs in vitro. In vivo results from a diabetic skin wound model showed that the hAAM-Exos dressing accelerated wound healing by regulating inflammation, stimulating vascularization, and promoting the production of extracellular matrix.

**Conclusion:**

Exosome-incorporated hAAM scaffolds showed great potential in promoting diabetic skin wound healing, while also providing strong evidence for the future clinical applications of ADSC-derived exosomes.

## Introduction

With the high prevalence of diabetes around the world, complications such as diabetic wounds have become a serious threat to the health and quality of life of patients [1]. Traditional clinical therapies for diabetic wounds include control of blood glucose, surgical debridement, negative pressure therapy, and graft transplantation [2–4]. However, these therapies are ineffective in many cases due to the particular impaired pathological conditions of the wound sites [5]. Therefore, new therapeutic approaches that will facilitate diabetic wound healing are urgently needed. In recent years, mesenchymal stem cells (MSCs) have been demonstrated to be a promising new therapy for diabetic wound healing [6–8], yet many challenges remain, such as immunological rejection and chromosomal variation, as well as ethical issues, which limit their clinical utility [9, 10]. It was once thought that transplanted MSCs could reside in damaged tissues and play a role in cell replacement through direct differentiation [11]. However, recently, emerging studies have shown that the beneficial effects of MSCs in vivo can be attributed to their paracrine action that regulates the functions of host cells and tissues [12–14]. Exosomes are nanosized membrane vesicles of endocytic origin, with a diameter of 30–150 nm, that are secreted by most cells [14]. It is well established that exosomes carry mRNAs, microRNAs, and proteins and are involved in cell-to-cell communication, cell signaling, and altering cell metabolism in vivo [15]. Many studies demonstrated that MSC-derived exosomes have similar biological functions to MSCs themselves and can be used as a possible therapy [16]. Recent studies of the application of MSC-derived exosomes for promotion of healing of chronic diabetic wounds have shown encouraging results [17–19]. Relative to other types of MSCs, adipose-derived mesenchymal stem cells (ADSCs) have special advantages such as their easy availability and rich supply [20]. Various studies indicated that ADSC-derived exosomes enhanced wound healing by regulating inflammatory responses, accelerating angiogenesis, increasing migration and proliferation of keratinocytes and fibroblasts, and activating collagen and elastin synthesis by fibroblasts [21–24]. Moreover, ADSC-derived exosomes (ADSC-Exos) also reduced scarring by regulating extracellular matrix (ECM) remodeling [25]. However, how to use exosomes in wound treatment effectively is a problem that needs to be addressed at present. In order to make the use of exosomes more realistic for clinical applications, a simple, effective, and noninvasive method is needed.

Wound dressings based on decellularized biomaterials, which, as natural materials, have received considerable research interest, are gaining popularity in regenerative medicine [26]. Specifically, human amniotic membrane is easily accessible and without ethical restrictions. Human acellular amniotic membrane (hAAM) has shown great potential as a scaffold for the repair of tissues and organs [27–29]. There have been many reports regarding the use of hAAM for wound coverage and use of composite hAAM combined with stem cells for the treatment of skin defects and functional repair [30–33]. Therefore, we hypothesized that hAAM may be one of the options to achieve delivery of exosomes directly to skin wounds, which may be more realistic for clinical application.

**Scheme 1:**
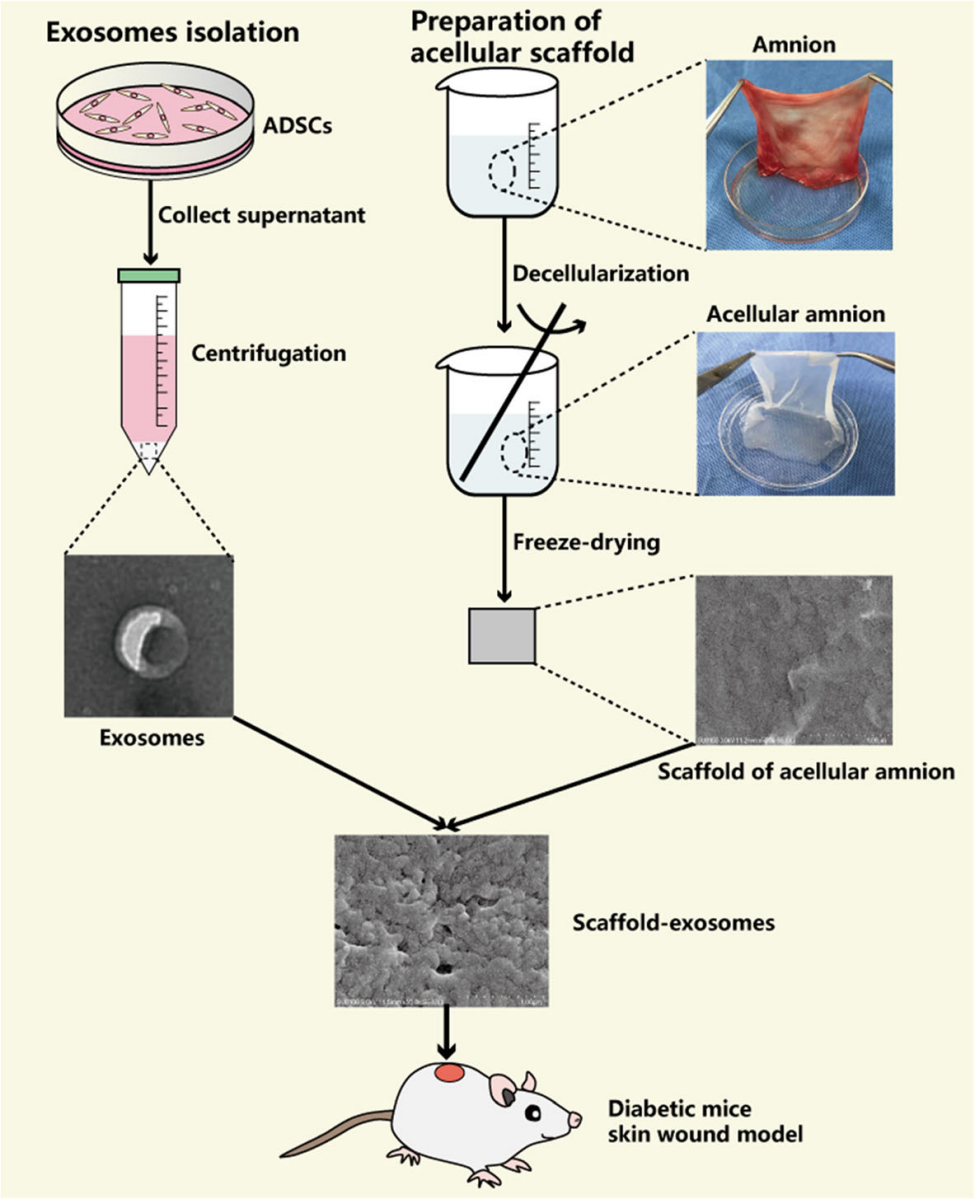
Schematic illustration of the isolation of adipose-derived mesenchymal stem cell (ADSC)-derived exosomes and preparation of hAAM scaffold for a diabetic mouse skin wound model

In this study, we aimed to improve wound healing in diabetic mice by externally applying a constructed combination of ADSC-Exos and hAAM (Scheme 1). After preparation and characterization of a scaffold of hAAM loaded with ADSC-Exos in vitro, they were transplanted onto wounds in vivo and wound healing phenomena were observed to identify the wound-healing mechanism of the exosome-hAAM composites.

## Materials and methods

### Animals and ethical approval

Eight-week-old BABL/C male mice were provided by the Experimental Animal Centre of Southern Medical University (Guangzhou, China). Human subcutaneous adipose tissue was obtained during liposuction procedures, and human amniotic membranes were obtained from healthy women undergoing a cesarean section in Nan Fang Hospital of Southern Medical University, after obtaining written informed consent. All protocols were approved by the Ethics Committee of Nan Fang Hospital of Southern Medical University. All animal experiments were approved by the Institutional Animal Care and Use Committee of Nan Fang Hospital of Southern Medical University and conducted according to the guidelines of the National Health and Medical Research Council (China).

### Isolation and culture of human ADSCs

Isolation and culture of human ADSCs were performed as previously described [20]. Briefly, the adipose tissue was harvested from the lower abdomen during liposuction procedures and digested with 0.075% collagenase type I (Sigma-Aldrich, St. Louis, MO, USA) for 45 min in a shaker incubator at 37 °C. After digestion, the adipose cell suspension was centrifuged at 800×*g* for 5 min, then the cell pellet at the bottom was resuspended in PBS and filtered through a 100-μm mesh cell strainer. After further centrifugation at 800×*g* for 5 min, the cell pellet was resuspended in Dulbecco’s modified Eagle’s medium (DMEM; Gibco) and cultured in DMEM supplemented with 10% fetal bovine serum (FBS; Gibco) and 1% penicillin-streptomycin (Gibco) at 37 °C with 5% CO_2_. At 80% confluence, ADSCs were subcultured and cells at passage 3–5 were used in the present study.

### Isolation and identification of ADSC exosomes

Exosomes were extracted using an exosome isolation kit (Invitrogen, Grand Island, NY, USA) according to the manufacturer’s protocol. Briefly, after ADSCs reached 60–65% confluence, the culture medium was replaced with DMEM supplemented with 10% exosome-free FBS (Cell Max, Beijing, China), and the cells were cultured for another 48 h. The supernatants were collected and centrifuged at 2000×*g* for 30 min and then passed through a 0.22-μm filter to remove dead cells and cellular debris. The cell-free culture medium was collected and exosome extraction reagent was added to the medium at a ratio of 1:2. The culture medium/reagent mixture was mixed well and incubated at 4 °C overnight. After incubation, the samples were centrifuged at 10,000×*g* for 1 h at 4 °C, and the pelleted exosomes were resuspended with PBS. The protein content of the exosome suspension was determined using a BCA quantitation kit (Solarbio, Beijing, China).

The morphology, size, and marker (CD81 and CD9) expression of ADSC-Exos were analyzed by transmission electron microscopy (TEM, JEM-1400, JEOL Ltd., Tokyo, Japan), nanoparticle tracking analysis (NTA) using a NanoSight NS300 (Malvern Panalytical, Malvern, UK), and western blotting, respectively. ADSC-exosomes were used for experiments or stored at − 80 °C.

### Preparation and characterization of hAAM

The hAAM was prepared using methods modified from our previously published protocol for placenta [34]. The amnion was separated from the chorion and subsequently frozen at − 80 °C for 48 h and then thawed prior to decellularization. To prepare the hAAM, the amnion was washed thoroughly with PBS to remove blood and cellular debris and then incubated in 0.25% EDTA at 37 °C for 30 min. After digestion, the epithelium was scraped off with a cell scraper. Membranes were then decellularized in devitalization buffer containing 0.25% *N*-ethylmaleimide (Sigma-Aldrich) and 2% *N*-lauryl sarcosine (Sigma) at room temperature. The devitalization solution was removed and replenished every 4 h, for a total of 12 h. After repeated washing in distilled water, the hAAM was dialyzed against water at 4 °C for 24 h to remove excess reagents. Both intact human amniotic membrane (hAM) and hAAM were fixed in 4% paraformaldehyde (Solarbio), and hematoxylin-eosin (HE) staining was performed to observe whether the cells present in hAM had been removed completely in hAAM. Following dialysis, decellularized hAAM was lyophilized overnight and subsequently sterilized overnight with ethylene oxide gas then stored at 4 °C.

The lyophilized hAAM was immersed in distilled water or PBS for 24 h at room temperature until it reached a swelling equilibrium state. The swelling degree was calculated as follows: degree of swelling = (weight of wet hAAM - weight of dry hAAM)/weight of dry hAAM × 100%. The moisture retention capacity of the hAAM was evaluated by the same method. Briefly, after achieving the swelling equilibrium state of hAAM, the wet hAAM was placed in a glass dryer at room temperature, and the changes in the swelling ratio were determined every 3 h.

The surface morphology and structure of the hAAM scaffold were evaluated by scanning electron microscopy (SEM, Hitachi, Tokyo, Japan; S4800). In addition, 100 μg of exosomes were resuspended in 100 μL PBS and loaded onto a circular piece of hAAM with a diameter of 1 cm. The presence of the exosome particles on the hAAM were then detected by SEM.

### Cell proliferation, migration, and tube formation assay

Human umbilical vein endothelial cells (HUVECs) and human dermal fibroblasts (HDFs) were all purchased from the Cell Bank of the Chinese Academy of Sciences (Shanghai, China). HUVECs were cultured in endothelial cell medium (Sciencell, San Diego, CA, USA) supplemented with 5% fetal bovine serum (FBS; Sciencell), 1% endothelial cell growth supplement (Sciencell), and 1% penicillin-streptomycin (Sciencell) at 37 °C with 5% CO_2_. HDFs were cultured in DMEM (Gibco) supplemented with 10% fetal bovine serum (Gibco) and 1% penicillin-streptomycin (Gibco) at 37 °C with 5% CO_2_.

For cell proliferation, HUVECs and HDFs were seeded, separately, at a density of 4 × 10^3^ cells/well in 96-well culture plates and 100 μL of the above cell culture medium was added to each well. The cells were stimulated with 25 μg (0.25 μg/μL), 50 μg (0.5 μg/μL), or 100 μg (1 μg/μL) ADSC-Exos for 1, 3, or 5 days. Cell proliferation was assayed using a Cell Counting Kit-8 (Sigma), and absorbance of culture media was measured at 450 nm using a multilabel counter (*n* = 3).

For the cell migration assay, HDFs were seeded at a density of 1.5 × 10^5^ cells/well in 6-well plates and cultured to 100% confluence. Five parallel scratches were then made in each well with a 200 μL pipette tip and 1 mL of medium was added to each well. The cells were stimulated with 250 μg (0.25 μg/μL), 500 μg (0.5 μg/μL), or 1000 μg (1 μg/μL) ADSC-Exos for 12 or 24 h. The migration rate was measured immediately and after 12 h and 24 h using ImageJ software.

Tube formation was measured using a Matrigel method. Briefly, a 60-μL Matrigel (BD Biosciences, San Jose, CA, USA) solution was added to each well of a 96-well plate and incubated at 37 °C for gel formation. After the HUVECs were serum-starved for 24 h, cells were inoculated into 96-well plates at a density of 2.5 × 10^4^ cells/well, and 100 μL of medium was added to each well. The cells were stimulated with 25 μg (0.25 μg/μL), 50 μg (0.5 μg/μL), or 100 μg (1 μg/μL) ADSC-Exos for 24 h, and the tube formation was observed with a reverse phase-contrast microscope (IX61 FL, Olympus, Tokyo, Japan).

### Streptozotocin-induced diabetic mice and wound closure assay

After 3 weeks of hyperglycemia (glucose level no lower than 16.8 mmol/L) induced by intraperitoneal injection of streptozotocin (150 mg/kg), a full-thickness skin defect model was generated. Diabetic BABL/C mice were anesthetized by intraperitoneal injection of 10 g/L pentobarbital sodium (0.4 mL/100 g), then the dorsum was shaved and cleaned with betadine. A circular full-thickness skin defect wound 1 cm in diameter was created on the back, and the defect area was fixed with a ring-shaped silicone. Mice were randomized into four groups. Control group: the wound was covered with a gauze circle (10 mm in diameter) containing 100 μL PBS (*n* = 10); Exosome group: the wound was treated with 100 μL PBS containing 100 μg exosome externally (*n* = 10); hAAM group: the wound was covered by a circular hAAM patch (10 mm in diameter) containing 100 μL PBS (*n* = 10); hAAM-Exos group: the wound was covered by a circular hAAM patch (10 mm in diameter) preloaded with 100 μL PBS containing 100 μg exosomes (*n* = 10). Afterwards, a piece of Vaseline gauze was used to cover the gauze (control group), the wound (exosome group), or the hAAM (hAAM and hAAM-Exos groups). Finally, Tegaderm™ (3 M, St. Paul, MN, USA) was used to fix the wound and dressings. The wound dressings in each group were changed every other day, three times in total according to the above methods. The mice were housed individually and wound healing was evaluated on the basis of gross observation at days 1, 3, 7, and 14 post-operation. The wound healing rate was calculated as follows: (primary wound size—residual wound size)/original wound size × 100%.

### Histological analysis

Wound areas including the surrounding skin were collected at 5, 7, 14, and 21 days after operation. The samples were fixed in 4% paraformaldehyde (Solarbio), gradually dehydrated, embedded in paraffin, and cut into 4 μm sections. The sections were stained with hematoxylin and eosin (H&E) or Masson’s trichrome stain according to the manufacturer’s instructions (Sigma-Aldrich). H&E staining was used to assess infiltration of inflammatory cells, and Masson’s staining was used to determine the content and maturity of collagen in the wound beds. The number of inflammatory cells per field in each group at day 5 and day 14 post-operation was calculated. Image-Pro Plus 6.0 software was used to calculate the collagen deposition at days 7 and 14 post-operation. Statistical analysis was performed based on five high-powered fields per sample.

### Immunohistochemical analysis

In order to further observe the wound inflammation, M2 macrophages were identified by immunohistochemical staining of CD206. To evaluate extracellular matrix (ECM) production and remodeling during the wound process, collagen expression was determined by immunohistochemical staining of collagen III. Angiogenesis was measured by immunohistochemical staining of CD31 in the wound bed. For immunohistochemical staining, the sections were treated with antigen retrieval and then incubated with primary antibody: CD206 (1:150, Abcam, Cambridge, MA, USA), collagen III (1:200, Abcam), and CD31 (1:200, Abcam) at 4 °C overnight. The sections were then incubated with secondary antibody (1:250, Abcam) for 30 min, stained with diaminobenzidine (Invitrogen) and hematoxylin, dehydrated, cleared, and mounted.

### Statistical analysis

Experimental data are expressed as the mean ± standard deviation. Comparisons between different groups were assessed by one-way analysis of variance (ANOVA). Differences with *P* < 0.05 were considered statistically significant.

## Results

### Characterization of hAAM scaffold

**Fig. 1:**
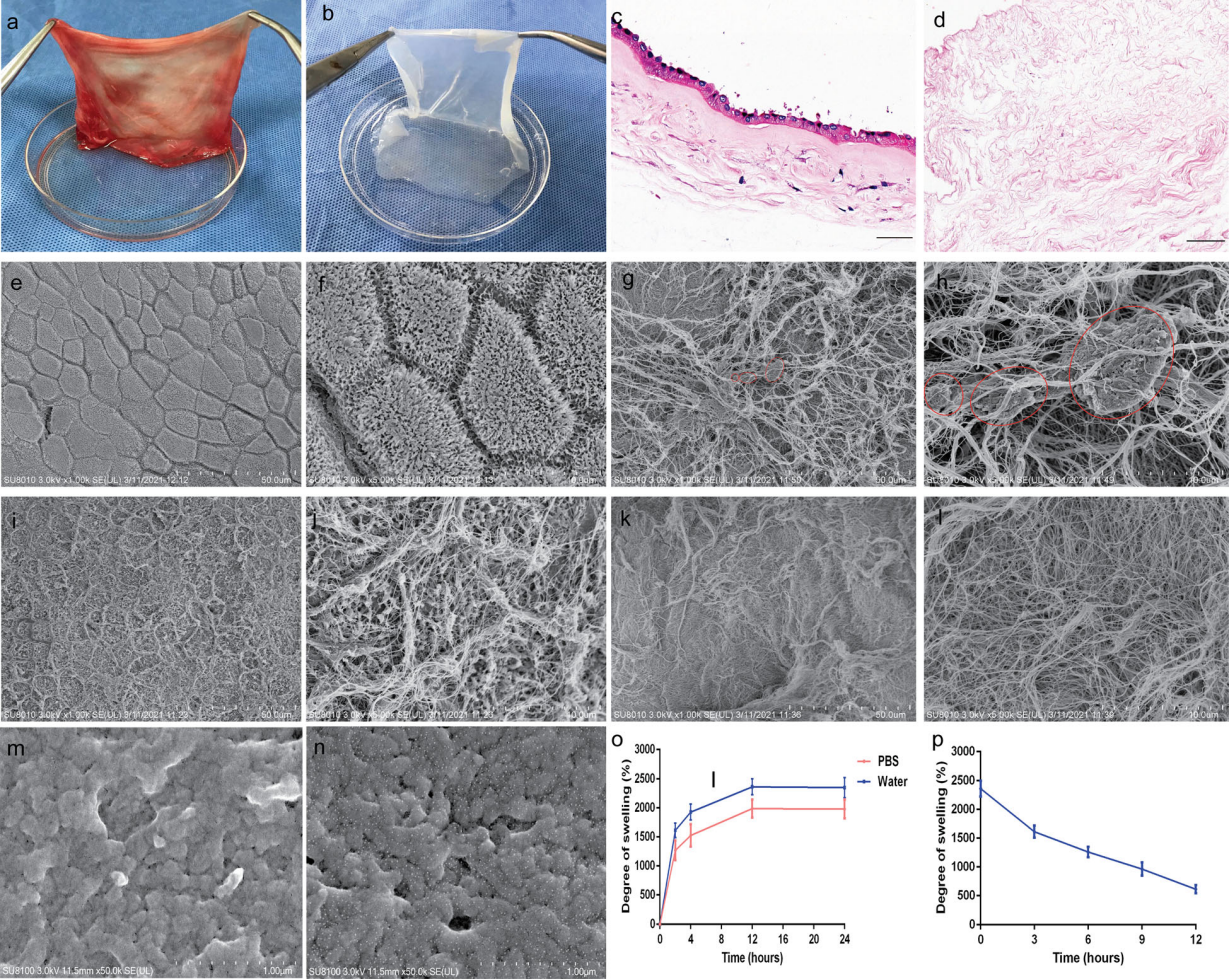
Characterization of human acellular amniotic membrane (hAAM). **a** The appearance of human amniotic membrane (hAM) tissue before decellularization. **b** The appearance of hAM after decellularization when the tissue became translucent. **c** Hematoxylin & eosin (H&E) staining of hAM before decellularization, showing the presence of a large number of nuclei (scale bar = 50 μm). **d** H&E staining of hAM after decellularization showing almost no cell residue (scale bar = 50 μm). **e**, **f** Microstructures of the hAM before decellularization under SEM showed that epithelial cells arranged in a flat paving stone-like appearance on the epithelial side (**e**: scale bar = 50 μm, **f**: scale bar = 10 μm). **g**, **h** Intertwined collagen fibers with a small amount of stromal cells (red circle) were observed on the stromal side of the hAM (**g**: scale bar = 50 μm, **h**: scale bar = 10 μm). **i**, **j** Microstructures of the hAAM after decellularization under SEM showed the epithelial cells were removed after decellularization, and cell niches were observed on the epithelia side of the hAAM (**i**: scale bar = 50 μm, **j**: scale bar = 10 μm). **k**, **l** A porous structure arranged by collagen fibers without stromal cells were observed on the stromal side of the hAAM (**k**: scale bar = 50 μm, **l**: scale bar = 10 μm). **m** SEM image of the swelling hAAM surface (scale bar = 1 μm). **n** SEM image of the swelling hAAM surface after adding exosomes (scale bar = 1 μm). **o** Swelling degree of the hAAM in different media at different time-points. **p** Moisture retention capacity of the hAAM

After decellularization, the hAM tissue appeared translucent and devoid of cells. The matrix structures of native hAM and hAAM were visualized by H&E staining and almost no residual cells were observed (Fig. 1a–d). The morphology of hAM before and after decellularization was observed by SEM. Native hAM includes epithelial side and stromal side. The epithelial cells of hAM arranged in a flat paving stone-like appearance (Fig. 1e and f). On the stromal side of the hAM, intertwined collagen fibers were observed, with a small amount of stromal cells (Fig. 1g and h). After decellularization, the hAAM revealed a three-dimensional porous structure arranged by collagen fibers, with many cell niches, and no obvious epithelial and stromal cells remained (Fig. 1i–l). After freeze-drying under vacuum, sterilization, and swelling, the porous morphology of the hAAM scaffold was also observed (Fig. 1m). After loading with exosomes, exosome particles could be observed on the hAAM (Fig. 1n).

The hAAM matrix exhibited good swelling properties and moisture retention capacity. The swelling properties of the hAAM in water or PBS at different time points are shown in Fig. 1o. After immersion for 12 h, swelling equilibrium was reached and the swelling ratio was 23 and 20 in water and PBS, respectively. In addition, the hAAM also exhibited good moisture retention capacity (Fig. 1p). The water retention time was more than 12 h and after 12 h the hydrogel contained 614% of its own weight in water residue.

### Characterization of ADSC-Exos

**Fig. 2:**
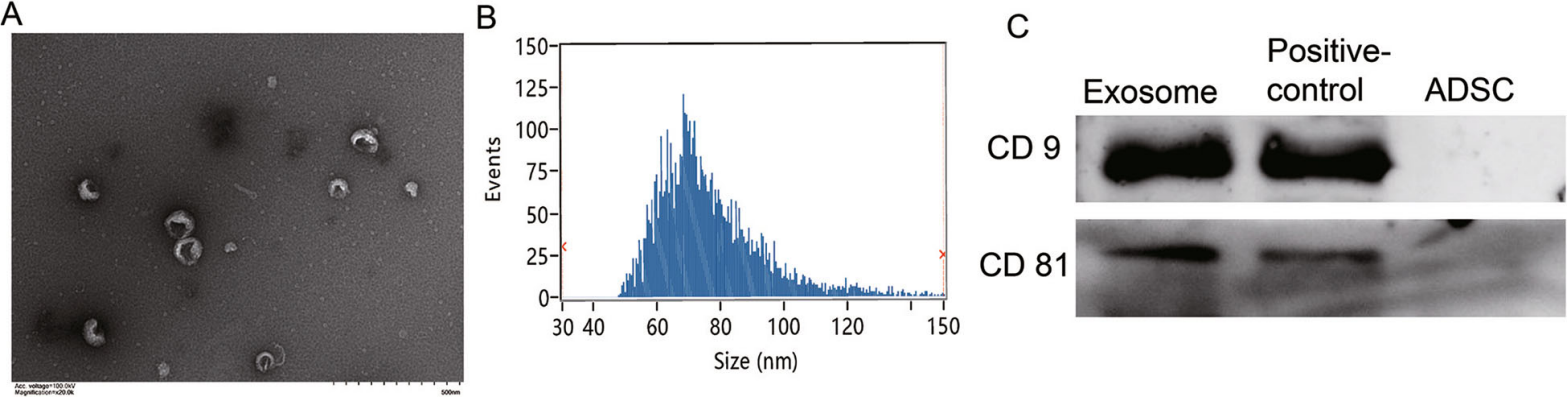
Characterization of ADSC-derived exosomes. **a** Representative images showing the morphology of ADSC-derived exosomes by transmission electron microscopy (scale bar = 500 nm). **b** NTA analysis demonstrating the diameter of exosomes which ranged from 47.7 to 150.0 nm, with a mean diameter of 76.4 nm. **c** Expression of exosomal markers (CD9 and CD81) examined by western blot analysis

The obtained exosomes derived from ADSCs were spherical with a closed membrane (Fig. 2a). The ADSC-Exos were assayed by NTA, and the diameter of the exosomes was shown to range from 47.7 to 150.0 nm, with an average diameter of 76.4 ± 16.48 nm (Fig. 2b). Exosome-specific surface markers (CD9 and CD81) were detected by western blotting (Fig. 2c).

### Effects of ADSC-Exos on human dermal fibroblasts (HDFs) and human umbilical vein endothelial cells (HUVECs) in vitro

**Fig. 3:**
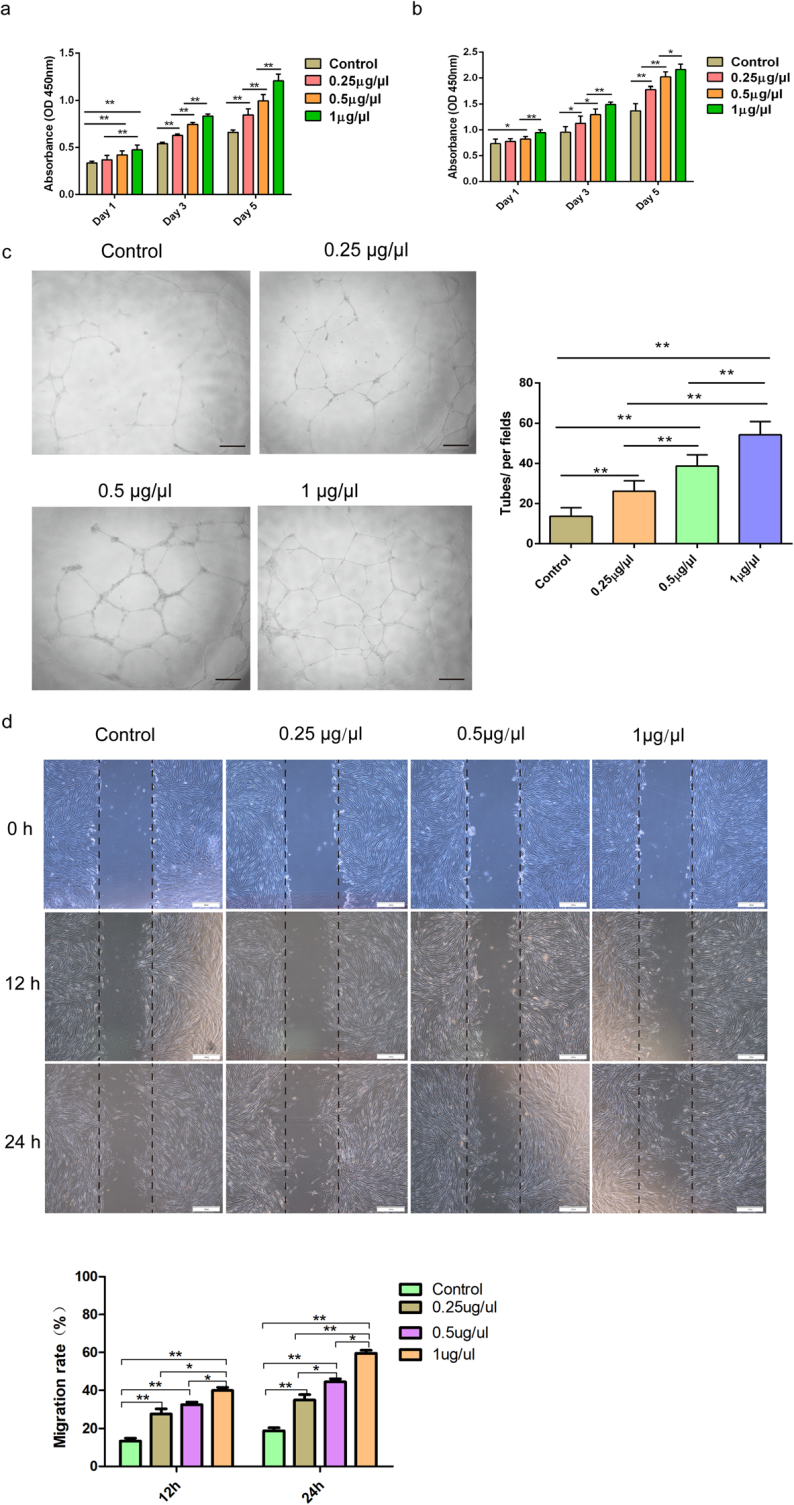
In vitro assessment of the effects of ADSC-derived exosomes on HUVECs and HDFs. Exosomes promoted the proliferation of HUVECs and HDFs in a dose-dependent manner. Exosomes promoted the tube-forming ability of HUVECs and the migration of HDFs also in a dose-dependent manner. **a** CCK-8 assay results of HUVECs. **b** CCK-8 assay results of HDFs. **c** Tube formation results of HUVECs after 24 h. Scale bar = 500 μm. **d** Cell migration results of HDFs at 12 and 24 h. Scale bar = 500 μm. **P < 0.01, *P < 0.05

The proliferation of HUVECs and HDFs cultured for 1, 3, or 5 days in medium containing ADSC-Exos is shown in Fig. 3a and b. Exosomes promoted the proliferation of HUVECs and HDFs in a dose-dependent manner. Tube formation assays using HUVECs (Fig. 3c) and scratch assay using HDFs (Fig. 3d) were used to evaluate the proangiogenic and pro-migration capacity of the ADSC-Exos, respectively. The results showed that ADSC-Exos promoted tube-forming ability of HUVECs and migration of HDFs also in a dose-dependent manner.

### The hAAM-Exos promoted wound healing

**Fig. 4:**
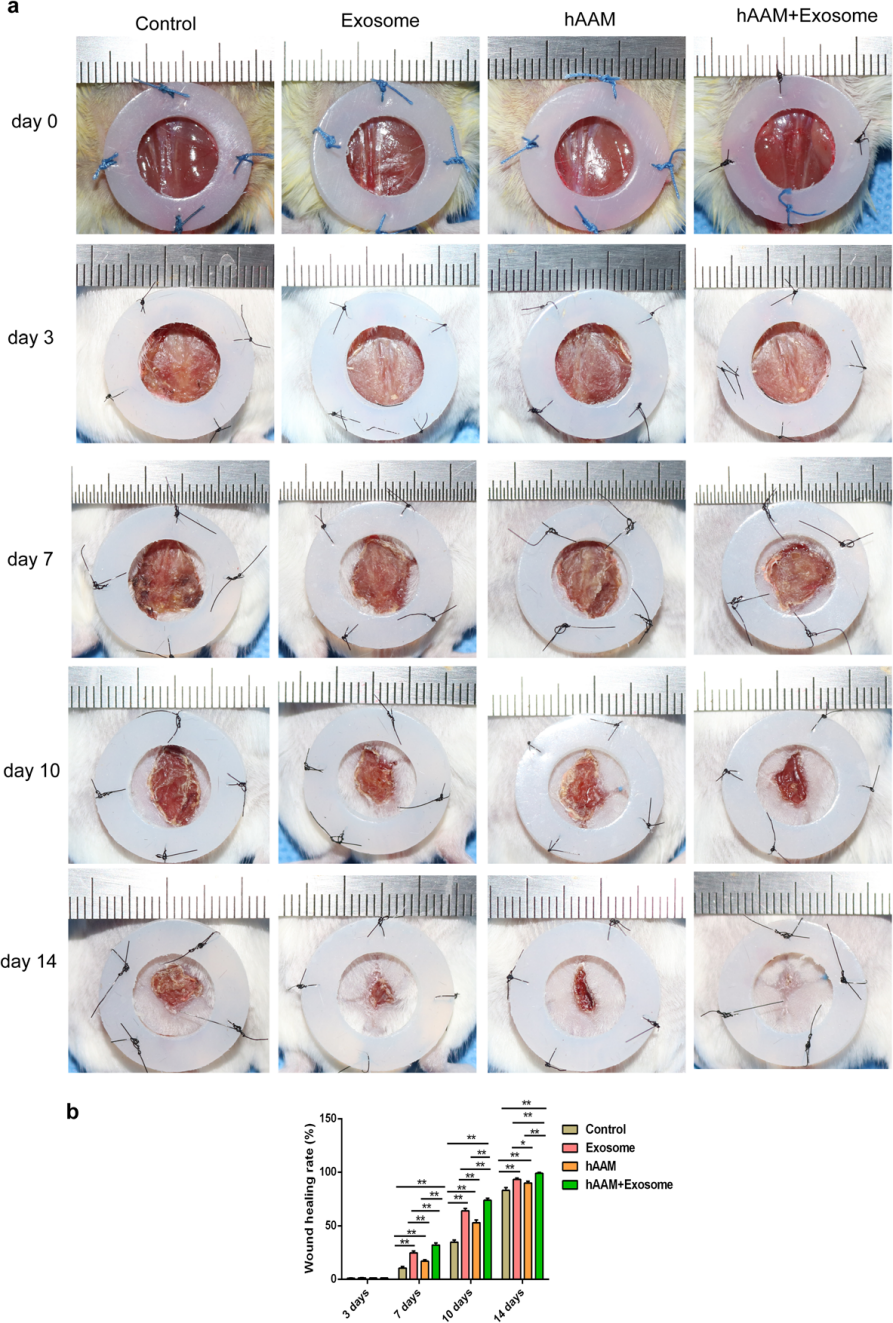
Healing process of diabetic wounds promoted by the hAAM-Exos scaffold dressing. **a** Representative images of the wound healing process in diabetic mice treated with control, hAAM, exosomes, or hAAM-Exos. **b** Wound closure rates at different time points of the four groups; **P < 0.01, *P < 0.05

The efficacy of hAAM-Exos for diabetic wound healing was investigated in diabetic mice. Figure 4a shows gross images of wounds at different time points. The wound sizes in all groups were obviously reduced after 7 days of treatment, among which the hAAM-Exos group showed more rapid healing than the others. Compared with the other three groups, the wounds in the hAAM-Exos group showed better healing appearance at day 14, with newly formed skin, whereas the other three groups still had unhealed wounds and the control group had the largest unhealed area. The wound closure rate also confirmed the gross observation results (Fig. 4b). The hAAM-Exos group showed the highest wound closure rate compared to the other three groups from day 7, followed by the exosome and hAAM groups.

### The hAAM-Exos exerted inflammation-regulating effects during the healing process

**Fig. 5:**
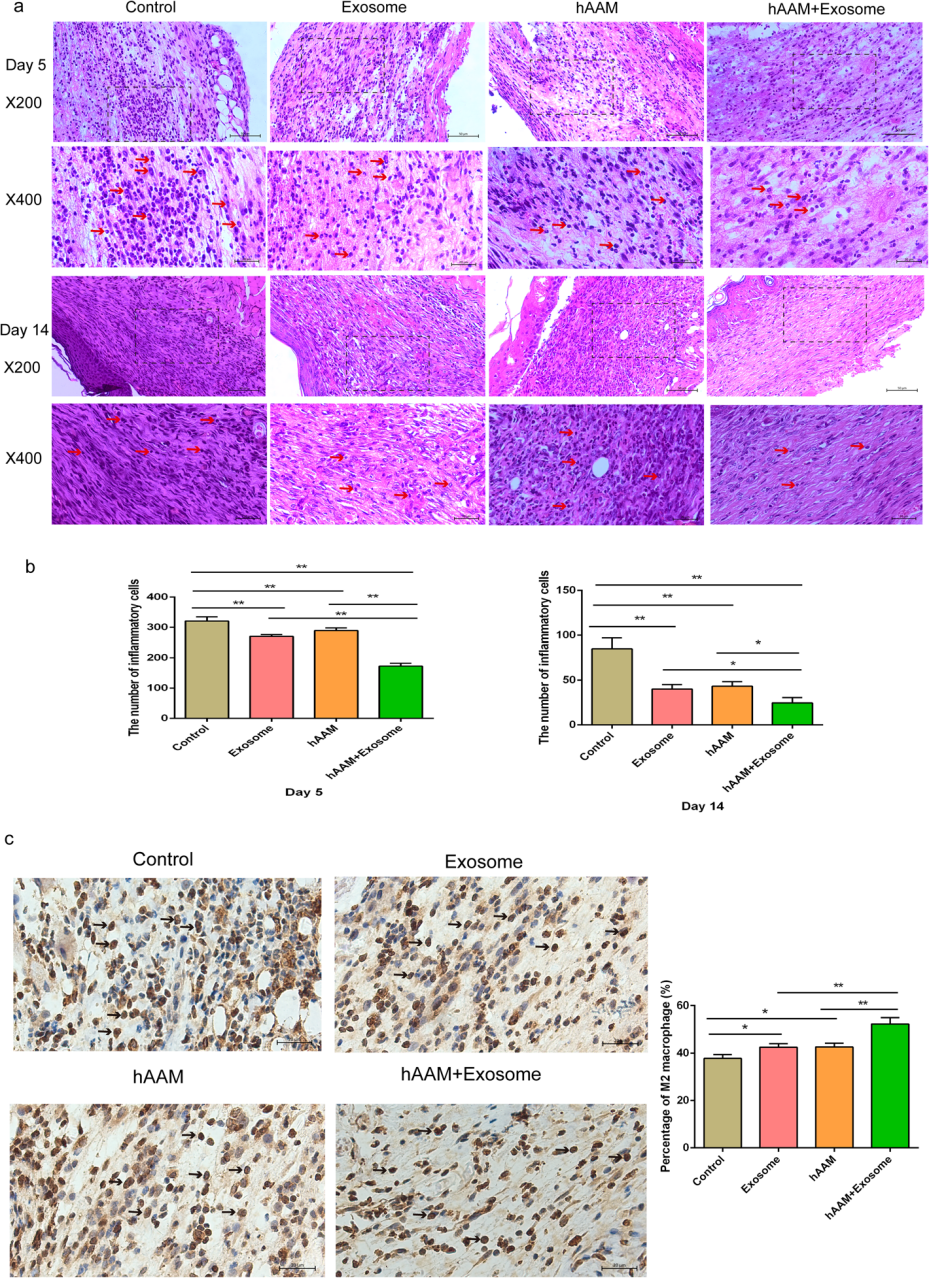
Infiltration of inflammatory cells during wound healing. The hAAM-Exos group showed the lowest number of inflammatory cells at days 5 and 14 and the highest proportion of anti-inflammatory cells (CD206+) at day 5. **a**, **b** Representative images and quantification results of inflammatory cells evaluated by H&E staining in all groups at days 5 and 14. The 200× image scale bar = 50 μm; the 400× image scale bar = 20 μm. **c** Representative images and quantification results of CD206+ cells by immunohistochemical staining at day 5. Scale bar = 20 μm; **P < 0.01, *P < 0.05

The hAAM-Exos suppressed wound inflammatory responses, compared with the control wounds and those treated with hAAM or exosomes. Figure 5a and b show the H&E staining results of inflammation in all groups at days 5 and 14. The hAAM-Exos group showed the lowest number of inflammatory cells at both time points, followed by the exosome and hAAM groups. M2 macrophages as anti-inflammatory cells are important players in tissue repair. Immunostaining of CD206^+^ cells revealed significantly higher recruitment of M2 macrophages to the wound sites in the hAAM-Exos group, followed by the exosome and hAAM groups at day 5 (Fig. 5c).

### The hAAM-Exos promoted vascularization

**Fig. 6:**
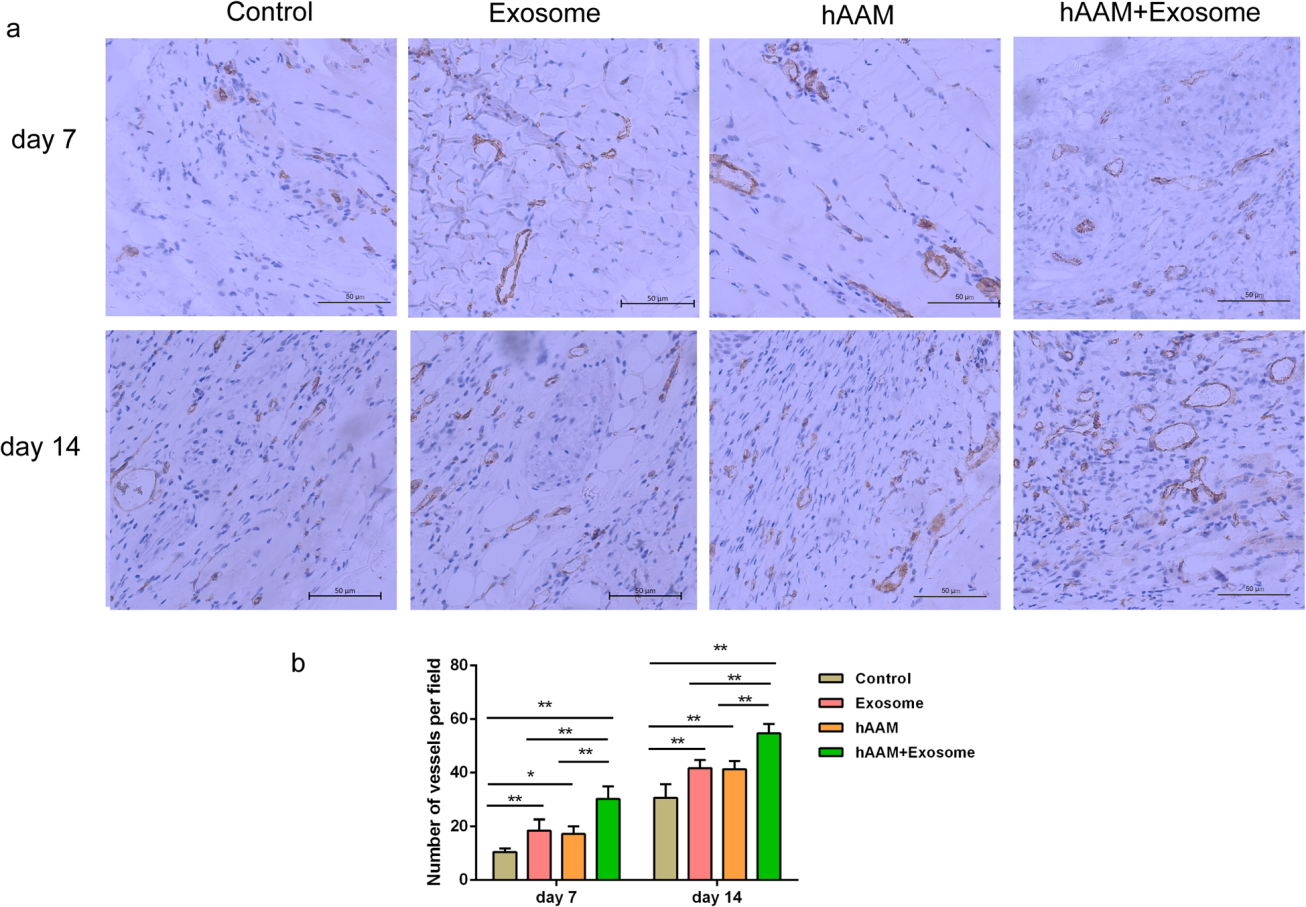
Immunohistochemical analysis of angiogenesis. The hAAM-Exos group showed the highest vessel numbers and densities. **a** Representative images of immunohistochemical staining of CD31 in each group at day 7 and day 14. Scale bar = 50 μm. **b** Quantitative analysis of the number of vessels per field in each group at day 7 and day 14. **P < 0.01, *P < 0.05

The hAAM-Exos scaffold dressing effectively promoted angiogenesis in diabetic wounds in vivo. Immunostaining of CD31 in vascular endothelial cells was performed at days 7 and 14 postoperation to investigate the proangiogenic effect of hAAM-Exos in vivo. At both time-points, few vessels were seen in either the control or the hAAM group; the exosomes group showed more regenerated vessels than these two groups, and the hAAM-Exos group showed the highest numbers and vessel densities (Fig. 6a and b).

### The hAAM-Exos enhanced extracellular matrix (ECM) deposition during healing

**Fig. 7:**
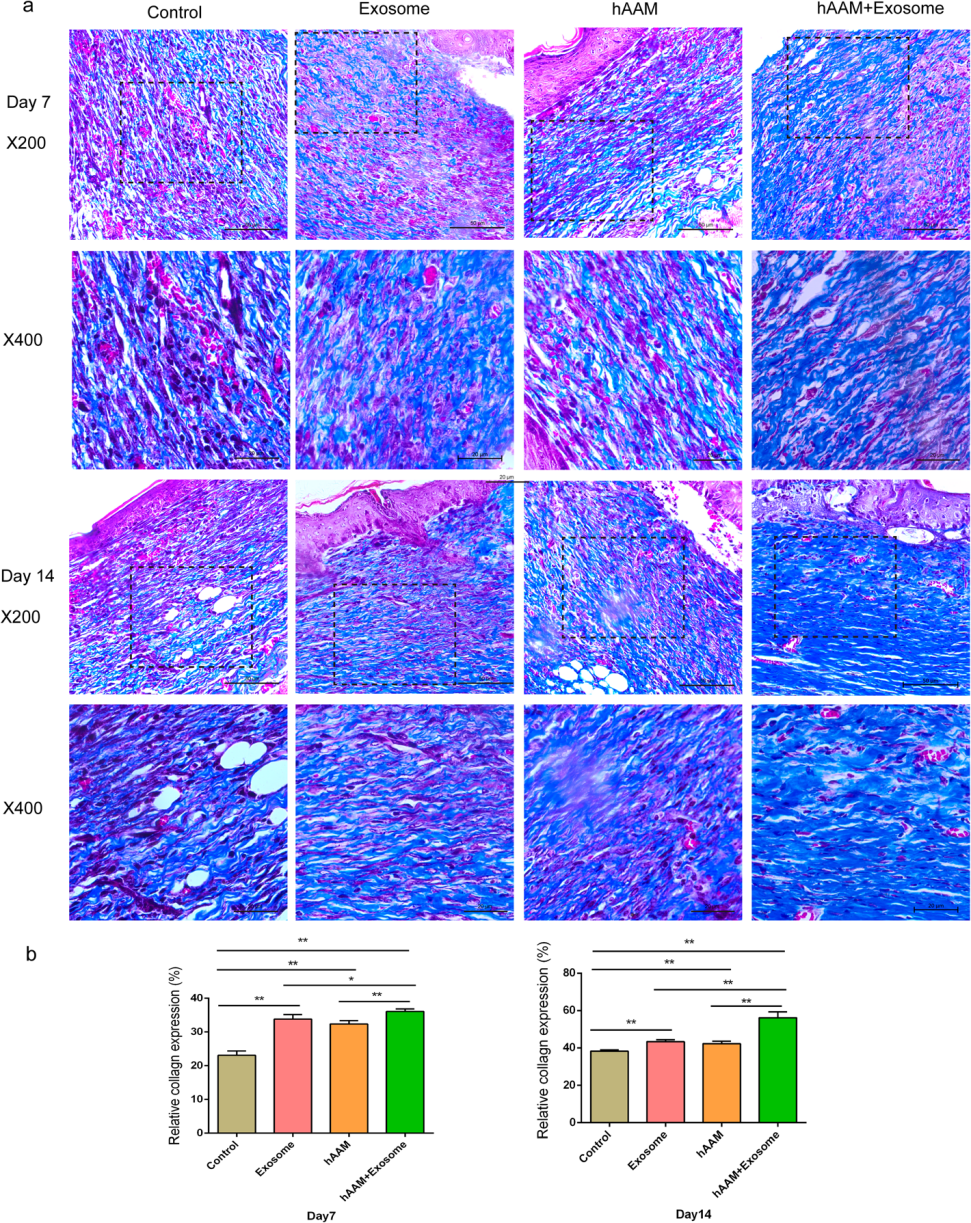
Regeneration of collagen was analyzed by Masson’s trichrome staining. The hAAM-Exos group showed the highest amount of deposited collagen compared to the other three groups. **a** Representative images of collagen evaluated by H&E staining in all groups at days 7 and 14. The 200× image scale bar = 50 μm, the 400× image scale bar = 20 μm. **b** Quantitative analysis of the collagen regeneration in each group at day 7 and day 14. **P < 0.01, *P < 0.05

To observe the production of ECM during the healing process, deposition of collagen was analyzed by Masson’s trichrome staining and immunostaining. Figure 7a and b show sections stained with Masson’s trichrome at days 7 and 14. At day 7, the hAAM-Exos group showed the highest amount of deposited collagen compared to the other three groups, followed by the exosomes and hAAM groups, while the control group showed the lowest collagen deposition. With the prolongation of healing time, collagen accumulation in all groups increased by day 14. More collagen accumulation was observed in the hAAM-Exos and exosome groups compared to the hAAM and control groups, and the highest collagen accumulation was observed in the hAAM-Exos group.

**Fig. 8:**
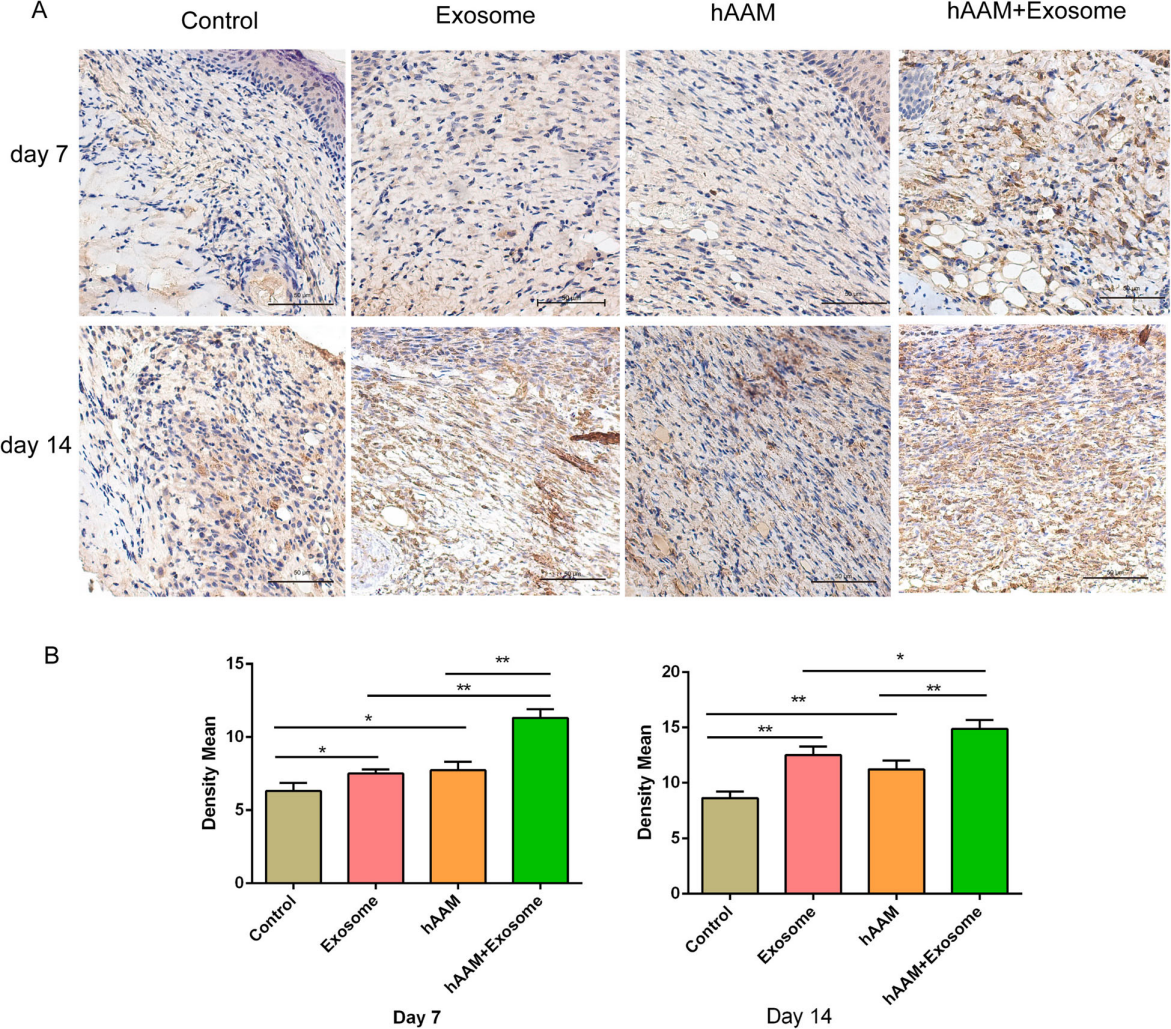
Regeneration of collagen was analyzed by immunostaining of collagen type III. **a**, **b** Representative images and quantification results of collagen type III evaluated by immunohistochemical staining at days 7 and 14. Scale bar = 50 μm; **P < 0.01, *P < 0.05

The results of immunostaining for collagen type III at days 7 and 14 are shown in Fig. 8a and b and similar trends to those seen with Masson’s trichrome staining were exhibited. With the prolongation of healing time, the deposition of collagens III also increased in all groups and the hAAM-Exos group showed the highest intensity compared to the other groups at 7 and 14 days.

### Regeneration of skin appendages during wound healing

**Fig. 9:**
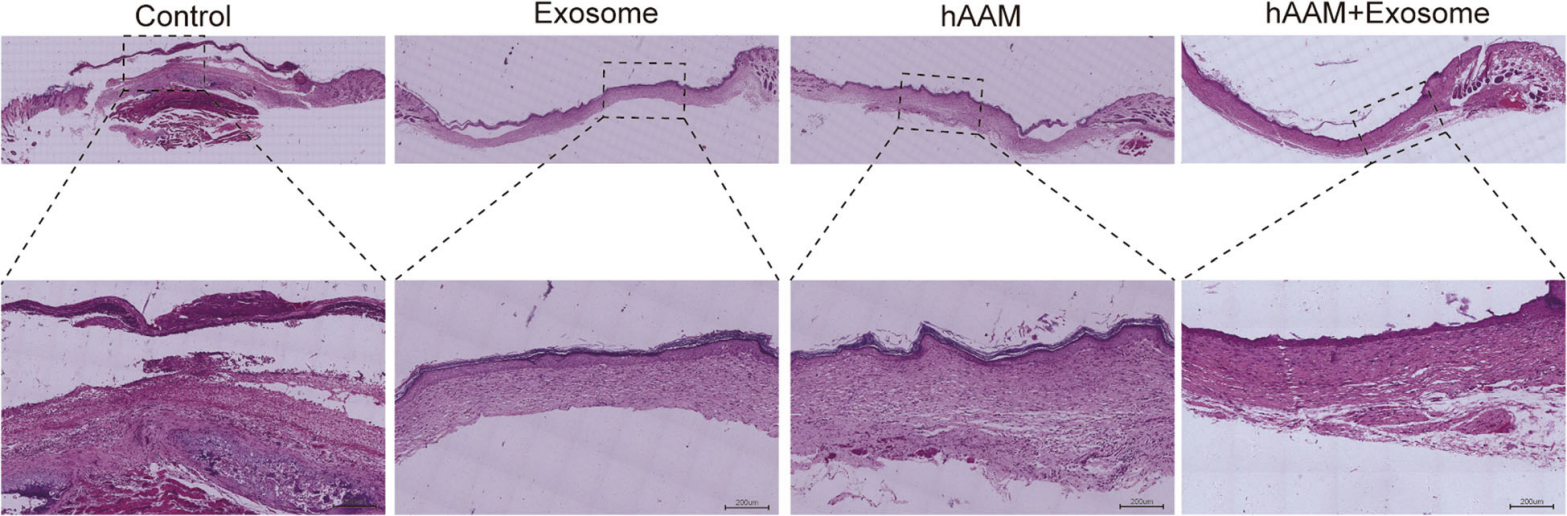
H&E analysis of the wound sites at day 21. The hAAM-Exos, pure exosome, and hAAM groups shown completed epithelial and dermal regeneration, while the control group exhibited no completely re-epithelialization. Hair follicle and sebaceous glands failed to regenerate in the wound sites in all groups, while mature hair follicles could be observed around the wounds. Scale bar = 200 μm

To examine the formation of skin appendages including hair follicles and sebaceous glands during wound healing, histological sections of the skin at the wound sites were performed at day 21. At day 21, the control group still had unhealed wounds, while the other three groups had finished re-epithelialization, with newly formed skin. As shown in H&E analysis (Fig. 9), unlike the control group, which exhibited with no completely re-epithelialization, completed epithelial and dermal regenerated in the wound sites of hAAM-Exos, pure exosome, and hAAM groups at day 21. However, hair follicle and sebaceous glands failed to regenerate in the wound sites in all groups.

## Discussion

In this study, we constructed a combination of hAAM and ADSC-Exos to promote diabetic wound healing. Our in vitro results showed that ADSC-Exos promoted the proliferation and migration of HDFs in a dose-dependent manner. Furthermore, ADSC-Exos also promoted proliferation and tube formation of HUVECs. In our in vivo study, hAAM-Exos accelerated diabetic wound healing by regulating inflammation, stimulating vascularization, and promoting the production of ECM.

ADSCs have received extensive attention in the field of cell therapy and regenerative medicine. On the basis of both in vitro experiments and preclinical studies, ADSCs have been used in different clinical fields. ADSCs have been reported to be effective in promoting skin wound healing, tissue repair, and bone regeneration [35]. In particular, ADSCs were reported to be effective in diabetic wounds [36]. The beneficial application of ADSCs combined with fat transplantation was also reported in soft tissue augmentation surgery, increasing the long-term maintenance of fat graft volume [37]. Moreover, ADSCs were used to improve hair regrowth caused by androgenic alopecia [38]. Additionally, recent studies reported that MSCs can exert their antiviral effects versus COVID-19, ADSCs, and ADSC-secreted exosomes could as a potential antiviral therapy [39]. ADSCs have a wide application prospect in the field of cell therapy and regenerative medicine, while the biomolecular mechanism involved is not yet clear and many challenges remain. Previous studies have found that the biological functions of MSCs stem from their paracrine actions and that the secretion of exosomes and microvesicles by MSCs are the main responsible for the therapeutic properties [14]. Corresponding studies on ADSC-derived exosomes have already shown their wide range of application prospects, including wound healing, tissue regeneration, tumors, and many other essential fields [40].

MSC-derived exosomes contain mRNAs, miRNAs, and proteins, which exert their biological functions through horizontal transfer to target cells [41]. The application of exosomes may provide a superior safety profile and avoid many risks associated with cell transplantation and thus have great potential to achieve “cell-free regenerative medicine” [14]. Previous studies have found that a large amount of micRNA was enriched in MSC-derived exosomes and facilitated the repair of wounds [41]. Baglio et al. [42] showed that the top five most abundant miR-RNAs in the ADSC-derived exosomes were miR-486-5p, miR-10a-5p, miR-10b-5p, miR-191-5p, and miR-222-3p. Zhu et al. [43] demonstrated that ADSC-derived exosomes contain a series of miRNA biological molecules, including miR-126, miR-130a, and miR-132, which have the ability to promote angiogenesis, as well as miR-let7b and miR-let7c which have anti-fibrotic properties. Yang et al. [44] found that miRNA-21 derived from ADSC-Exos promoted the migration and proliferation of keratinocytes and enhanced the synthesis and remodeling of collagen.

Although exosomes exhibit great potential in wound healing, realistic ways of applying the exosomes in the clinic are urgently needed. Direct contact with the wound site may have greater beneficial effects, but exosomes used alone directly on the wound are easily lost, reducing the therapeutic effect. Recent studies have reported the use of various types of hydrogel scaffolds loaded with MSC-derived exosomes and then applied directly to diabetic wounds, which achieved satisfactory healing results [18, 21, 45]. Thus, such biomaterial-based exosome therapy may represent a new therapeutic approach for wound healing. HAAM as an attractive biomaterial for wound healing is inexpensive and readily available. In clinical trials, hAAM as a wound coverage has shown excellent therapeutic effects [30, 31]. In addition, stem cells delivered with hAAM have been reported as a new approach to improve their regenerative effects [32, 33]. However, hAAM incorporating exosome for wound healing was not reported. In this study, we first reported applying hAAM, which acts not only as a wound dressing, but also as a non-invasive method of delivering exosomes directly to the wound. The hAAM showed good swelling and moisture-retention properties. A moist environment is essential for re-epithelialization of wounds [46]. Besides, SEM images showed that the exosome particles were well adhered to the hAAM scaffold, suggesting that hAAM may be a suitable scaffold for wound dressing and exosome delivery.

Wound healing is a complicated and dynamic process that consists of three major over lapping stages: inflammation, proliferation, and remodeling [47]. Excessive inflammation will result in delayed wound healing or even non-healing [47]. Exosomes derived from MSCs have immunosuppressive and immunomodulatory properties and can accelerate wound healing and reduce scar formation [48]. During the wound healing process, macrophages play critical roles, from contributing to the initiation of inflammation to eliminating pathogens, resolving inflammation once the pathogens are cleared, and initiating and maintaining tissue repair and regeneration [49]. As is well-known, the macrophage lineage is highly plastic [50]. The transition from an inflammatory phenotype (M1) to a wound healing/profibrotic (M2) phenotype induces progression from the inflammation phase to the tissue repair phase [50]. In our study, the hAAM-Exos group exhibited decreased inflammation with the lowest inflammatory cell infiltration. Moreover, more M2 macrophages were present in the hAAM-Exos group than in the other three groups at day 5. These data suggested that hAAM with incorporated exosomes can reduce inflammation and accelerate the healing process in diabetic wounds.

The proliferative phase of wound healing (the development of granulation tissue) is characterized by extensive fibroblast proliferation and ECM accumulation, providing suitable conditions for re-epithelialization [47]. Collagen type III is the predominant collagen type in the healing wound, which is the major component of granulation tissue [51, 52]. In addition, extensive angiogenesis also occurs at this stage [47]. It has been speculated that exosomes derived from ADSCs could be internalized by dermal fibroblasts, stimulating their migration, proliferation, and collagen synthesis [53]. In our in vitro study, we found that ADSC-Exos promoted the proliferation and migration of HDFs and enhanced the proliferation and tube-forming ability of HUVECs. These results are consistent with previous studies [23, 54]. In our in vivo study, we found that the hAAM-exosome group exhibited more collagen deposition, as evidenced by Masson’s trichrome staining, and stronger immunostaining of collagen III at both days 7 and 14. Moreover, a higher microvessel density was observed in the wound bed of the hAAM-Exos group. These results indicated that the ADSC-Exos loaded onto the hAAM scaffold dressing were able not only to maintain their function in vitro but also to play an important role in the repair of diabetic wounds in vivo. Thus, the hAAM scaffold was able to deliver exosomes directly to the skin wound, which resulted in a better therapeutic effect, and may be more realistic in clinical applications. However, further understanding of the molecular mechanisms is necessary. In addition, for successful clinical translation in the next few years, it will be necessary to emphasize standardized experimental protocols, detailed methodological reports, and clear definitions of exosome-based therapeutic products. Wound healing in adult mammals results in scar tissue lacking skin appendages. Although scar formation can meet the basic functional requirements of the skin in preventing infection and dehydration, it is undesirable and unfavorable. The regeneration of skin appendages and redevelopment of fully functional skin is the ultimate goals of skin wound healing.

## Conclusion

Our data showed that a hAAM scaffold dressing was very suitable for the delivery of exosomes. ADSC-Exos enhanced the proliferation and migration of HDFs and promoted proliferation and tube formation by HUVECs in vitro. In vivo, hAAM-Exos exerted effects of inflammation regulation, stimulating vascularization, and promoting the production of ECM, thereby accelerating the healing of diabetic wounds. Thus, our exosome-incorporated hAAM scaffold showed great potential for promoting diabetic wound healing. The mechanistic insights are insufficient is the limitation of the manuscript. Although further investigation is necessary, our results also provided strong evidence for the future clinical applications of ADSC-derived exosomes.

## Data Availability

The data used to support the findings of this study are included within the article.

## Abbreviations

MSCs: Mesenchymal stem cells
ADSC: Adipose-derived stem cell
ADSC-Exos: ADSC-derived exosomes
HUVECs: Human umbilical vein endothelial cells
HDFs: Human dermal fibroblasts
hAM: Human amniotic membrane
hAAM: Human acellular amniotic membrane
NTA: Nanoparticle tracking analysis
ECM: Extracellular matrix
